# Uniform Local Binary Pattern Based Texture-Edge Feature for 3D Human Behavior Recognition

**DOI:** 10.1371/journal.pone.0124640

**Published:** 2015-05-05

**Authors:** Yue Ming, Guangchao Wang, Chunxiao Fan

**Affiliations:** Beijing Key Laboratory of Work Safety Intelligent Monitoring, School of Electronic Engineering, Beijing University of Posts and Telecommunications, Beijing 100876, P.R. China; Beijing University, CHINA

## Abstract

With the rapid development of 3D somatosensory technology, human behavior recognition has become an important research field. Human behavior feature analysis has evolved from traditional 2D features to 3D features. In order to improve the performance of human activity recognition, a human behavior recognition method is proposed, which is based on a hybrid texture-edge local pattern coding feature extraction and integration of RGB and depth videos information. The paper mainly focuses on background subtraction on RGB and depth video sequences of behaviors, extracting and integrating historical images of the behavior outlines, feature extraction and classification. The new method of 3D human behavior recognition has achieved the rapid and efficient recognition of behavior videos. A large number of experiments show that the proposed method has faster speed and higher recognition rate. The recognition method has good robustness for different environmental colors, lightings and other factors. Meanwhile, the feature of mixed texture-edge uniform local binary pattern can be used in most 3D behavior recognition.

## Introduction

Human behavior recognition has been a hot topic for decades. It has a wide variety of applications, for example video monitoring, virtual reality and intelligent control. Over the past several decades, considerable efforts have been devoted to 2D human behavior recognition. As a result, accuracy for 2D human behavior recognition has been substantially improved [1, 2]. However, 2D human behavior recognition are still difficult because of the inherent flaws, such as the recognition rate being reduced greatly due to the factors as lighting variations, shadow or shaking, etc.

Due to the deficiency for using only the RGB videos as the 2D human behavior recognition algorithms do, the lowered cost of 3D acquisition devices, such as Kinect and Leap Motion, makes a strong push for performance improvement by adding 3D depth information. Hence, a wide range of algorithms have been proposed with RGB-D data. RGB-D videos can capture different shape and distance variations, which preserves more discriminative information. The distinctive advantages of 3D human behavior recognition compared with its corresponding RGB videos have improved the effectiveness of recognition and raises its accuracy [3, 4].

Though great strides have been made in 3D human behavior recognition, it is still challenging to obtain reliability in behavior recognition, particularly with complicating factors and environments. Especially, accurate motion object extraction and effective feature extraction are two of the crucial but unsolved issues.

Firstly, traditional motion object extraction based on RGB-D videos methods only depend on the geometric relationship between the object and the camera and do not incorporate motion trend between adjacent frames. Therefore, they have difficulties in handling the irrelevant backgrounds.

Secondly, traditional 3D human behavior features, such as HoG3D [5], 3D MoSIFT [6], etc., can only represent the surface features and are not able to comprehensively reflect the high-level contents of behavior videos. However, in real-world applications, high-level contents can better capture the discrimination information of behaviors. Moreover, traditional methods usually suffer the problem of high computation complexity.

**Fig 1:**
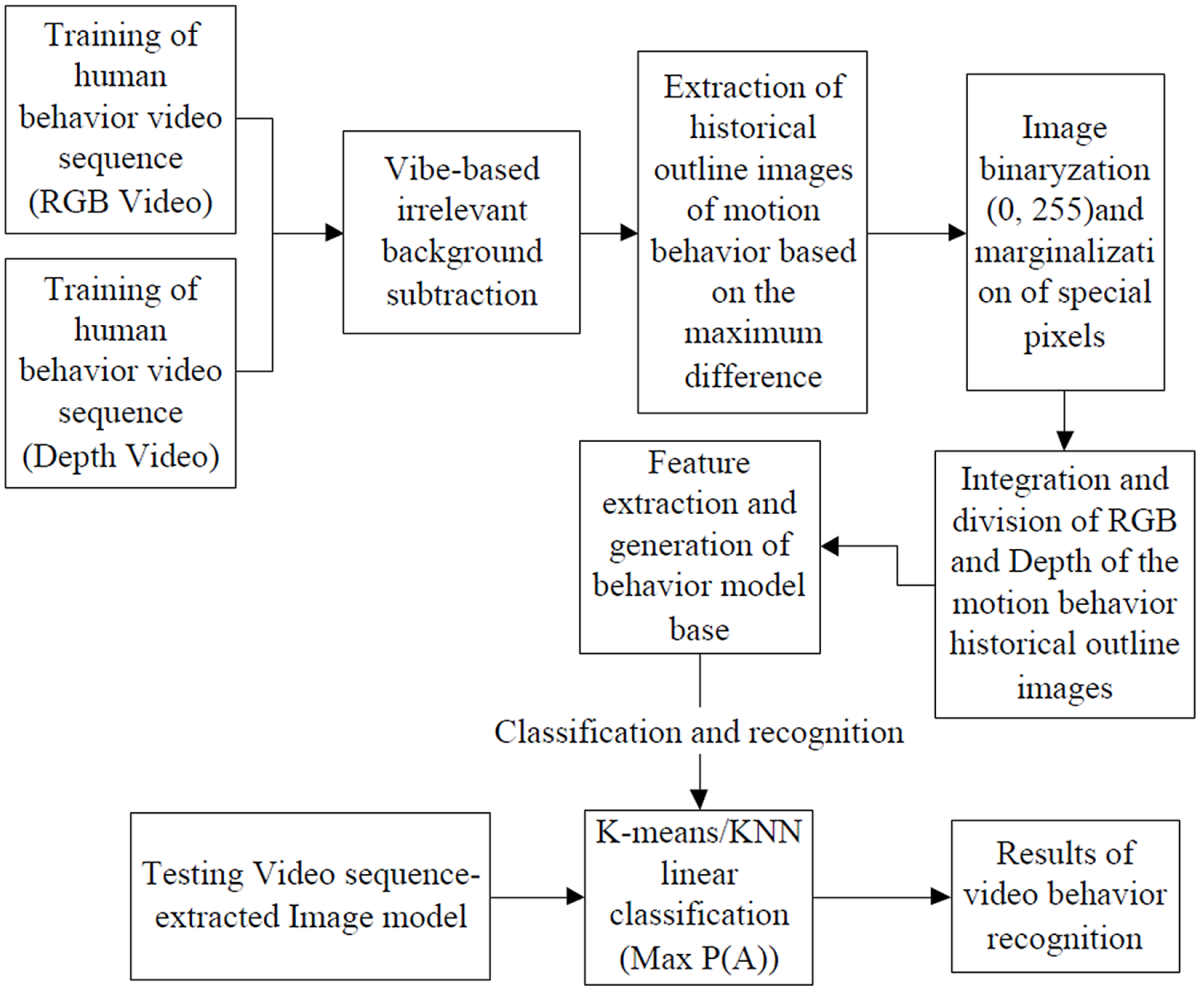
The Flow Diagram of the Process of Recognition Method and Mechanism.

The research in this paper focuses on the improvement and optimization of previous methods in feature extraction and behavior modeling. First, we use human behavior video sequences to conduct the background subtraction of training part, and extract the behavior outline historical images from the background subtracted images to acquire the depth and RGB images, which can represent the behavior videos. After that, the edge can be processed for depth and RGB images respectively. Then, the depth and RGB images will be blended together to obtain the outline historical image of human behavior, which can represent the videos. Therefore, a trained behavior model can be obtained after the feature extraction and classification. The flow diagram of the process is as shown in Fig 1.

The main contributions of this paper are summarized in the following items:

1. Reliability: We combine a modified ViBe (Visual Background extractor) and the historical image of behavior outline for motion object extraction. Different from previous research, the logic relationship and variation trend between the logic pixels of the frame images before and after the current frame is utilized to carry out inter-frame differential algorithm, which achieves excellent effects on the subtraction of irrelevant background.
2. Effectiveness: We derive a descriptor named uniform local binary pattern based texture-edge feature for 3D human behavior recognition. A hybrid texture-edge local pattern coding feature extraction and integration of RGB and depth videos lays profound foundation for the higher level data analysis in our practical applications of human behavior recognition.
3. University: The performance improvement of our 3D human behavior recognition framework which has demonstrated on different databases, where 3D human behavior videos were collected at various times, different environments, and from individuals from different countries, and with a large range of colors, lightings, poses, and complex backgrounds.

The main structure of this paper is carried out around the flow diagram. Related work about human behavior recognition is described in Section 2. Section 3 mainly introduces the preprocessing of behavior videos and images. Section 4 mainly proposes the features extraction and classification algorithms. Meanwhile, the mixture Texture-Edge Uniform Local Binary Pattern is being raised. Finally, the experiments based on behavior datasets are carried out and the recognition results are analyzed in Section 5.

## Related work about human behavior recognition

Previous studies on human behavior recognition mainly focus on RGB video behavior recognition. As early as 1994, Polana and Nelson [7] conducted the human motion behavior recognition by using the features of 2D grid for tracking human motions. Then, Bobick and Campbell [8] used MEI (Motion Energy Images) and MHI (Motion History Images) to present the human behaviors in image sequences. In 2003, Laptev et al. [9] raised up the idea of using Harris corner detector to detect the interest points in space-time 3D space and describe the related motions with the points. Y. Wang et al. [10] completed the recognition of human behavior by the classification of semi-latent topic models. Wu et al. [11] realized human action recognition with multi-modal feature selection and fusion. Recently, Moghaddam et al. [12] applied different training Initialization methods of Hidden Markov Models to human behavior recognition. Losifidis et al. [13] applied non-linear class-specific data projection to a discriminant feature space with applications in human behavior analysis. Fradi and Dugelay [14] focused on crowd behavior in public areas and proposed sparse feature tracking for crowd detection and event recognition. However, human behavior recognition based on RGB videos lacks depth information. It is difficult to reflect the distance and shape information, which results in the lower recognition rate.

With the rapid development of 3D motion sensing technology, researchers have gradually explored the characterization methods of the 3D videos about human behaviors. For the depth videos, Lin et al. [15] divided the depth sequences into space-time volumes and extracted features partially to realize the behavior recognition with ASM (Approximate String Matching). Ni et al. [16] proposed human action detection and recognition framework which was based on multi-stage depth-induced contextual information. Megavannan et al. [17] used depth MHI (Motion History Image) and its transformers to capture the motion change process and employed Hu moments to represent the features. Then, SVM classifier was used to recognize human behaviors. J. Wang et al. [18] achieved better effects on human behavior based on depth videos with a novel action-let ensemble model. Our previous work [19] developed 3D Mesh MoSIFT feature descriptor for human activity recognition based on the RGB-D video dataset, which demonstrated the superior performance for behavior analysis. Jalal and Kamal [20] presented a real-time life logging systems via depth silhouette for smart home services, which can provide monitoring, recording and recognition of daily human activities. Liu et al. [21] utilized a dynamic Bayesian network system to accurately estimate of human body orientation. During the studying process of depth video behavior recognition, the limitations of RGB videos are broken through [22]. But human behavior features merged with 3D depth information are more comprehensive than traditional 2D image information. As a result, most of the preset behavior algorithms and recognition mechanisms are tedious with poor robustness and low recognition rate.

The major relevant works of this paper are the irrelevant background subtraction and feature extraction. We will introduce the related work of these issues in the following subsections.

### Motion object extraction from the irrelevant background

There are two kinds of devices for depth information acquisition: the depth camera and stereo camera. The most representative of the depth camera might be the Kinect developed by Microsoft Corp. Kinect measures the distance in depth by infrared, which has the advantage in real-time situations. However, it is influenced by sunshine and complex backgrounds so that its detective range is limited. As a result, a huge number of methods for motion object extraction from the irrelevant background are proposed for RGB-D videos. Chattopadhyay et al. [23] used a hierarchical classification to realize motion feature extraction from incompleted RGB-D sequences. Li Jianfeng and Li Shigang [24] introduced eye-model-based gaze estimation to localize the head motion by RGB-D camera. Furthermore, the processing of RGB and depth videos includes ViBe (Visual Background extractor), which is used to subtract of irrelevant background, the marginalization of special local pixels and binarization of global pixel values. Barnich et al. [25, 26] has successfully applied ViBe foreground detection to video sequences and obtained good results.

However, the above mentioned methods do not incorporate the behavior outline model. Therefore, it can only coarsely extract the motion parts of human behaviors, which have less descriptive for fine depth variations.

### Feature extraction of 3D human behaviors

Because of the aforementioned advantages, also with the rapid advent of new capturing devices, the 3D human behavior recognition has triggered increased interest. The feature extraction of video’s outline model historical images is the critical process of behavior recognition [27]. There are many feature extraction algorithms of texture. As early as 2007, Hafiane et al. [28] put forward the texture classification algorithm of mid-value and binary value. Guo et al. [29] presented the texture classification algorithm of adaptive local binary value patterns. Liao [30] et al. created the explicit local binary patterns. Wang [31] et al. proposed a novel human detection approach based Histograms of Oriented Gradients and Local Binary Pattern (HOG-LBP). As for the application of human face recognition, Local Binary Patterns from Three Orthogonal Planes (LBP-TOP) was proposed by Zhao et al. [32] and Rotation Invariant Volume Local Binary Patterns (VILBP). The Local Ternary Patterns (LTP) created by Tan et al.[33] have good recognition performance. In the field of object detection, Amit et al. [34] published extended patterns of local binary and ternary patterns as Discriminative Robust Local Binary Pattern (DRLBP) and Ternary Pattern (DRLTP), which have greatly improved the efficiency of texture recognition. We draw on the experience of various local binary pattern algorithms to carry out texture extraction of the outline model historical images of human behavior.

However, it is far from enough to merely apply texture and margin features for feature extraction of behavior video. Texture and margin features can only represent the surface features, and they are not able to comprehensively reflect the high-level contents of behavior video. Therefore, the internal nature of behavior video should be mapped to its surface features, namely, to extract video’s frame images of its time-space history behavior.

## Preprocessing of human behavior video

Currently, the 3D depth video collection equipment mainly refers to Microsoft kinect. This equipment is mainly through the infrared devices to detect the distance between target users and the cameras without the need for pre-calibration of image. Different distance image regions can utilize different gray value description. During the preprocessing, we first apply ViBe motion object detection method to remove the irrelevant background while the behavior of human itself remains. Then, the motion history images of the depth and RGB videos are generated. We use the property that the depth video is less sensitive to depth distance and mix the MHI together to dissolve the noise that generated by the shake of clothes and environment circumstance. We process and divide the edge of binary image for further removing. Finally, we generate the MHI that fuse depth and color images for preparation of feature extraction. Thus, the whole preprocessing of RGB and depth behavior videos in this section mainly contains ViBe subtraction of background, marginalization of local pixels and binaryzation of global pixel values.

### Video preprocessing and motion outline history image extraction

We use the improved ViBe method for irrelevant background subtraction. In ViBe model, we usually set pixel (*x*, *y*) as center of the circle and randomly choose *N* pixels in the radius of *R* for initializing the background model throughout the experiment. In this paper, we assume *R* = 12 and *N* = 20. In the same time, we apply the conservative update strategy and foreground counting method. If one pixel is detected *T* times as foreground consecutively, then update the pixel as new background pixel. Here, we assume *T* = 20. In order to achieve fast computation and removal of Ghost area, we set the update probability of neighbor pixel model to 1/16.

Then, we conduct the binaryzation of background and foreground pixel points.

$$\begin{array}{c} Pix_{n}\left(x,y\right)\;=\{\begin{array}{c} \;\;\;0,\;\;\;(x,y)\;\in \;\;Background\;pixel\\ 255,\;(x,y)\;\in \;\;Foreground\;pixel \end{array} \end{array}$$ (1)

**Fig 2:**
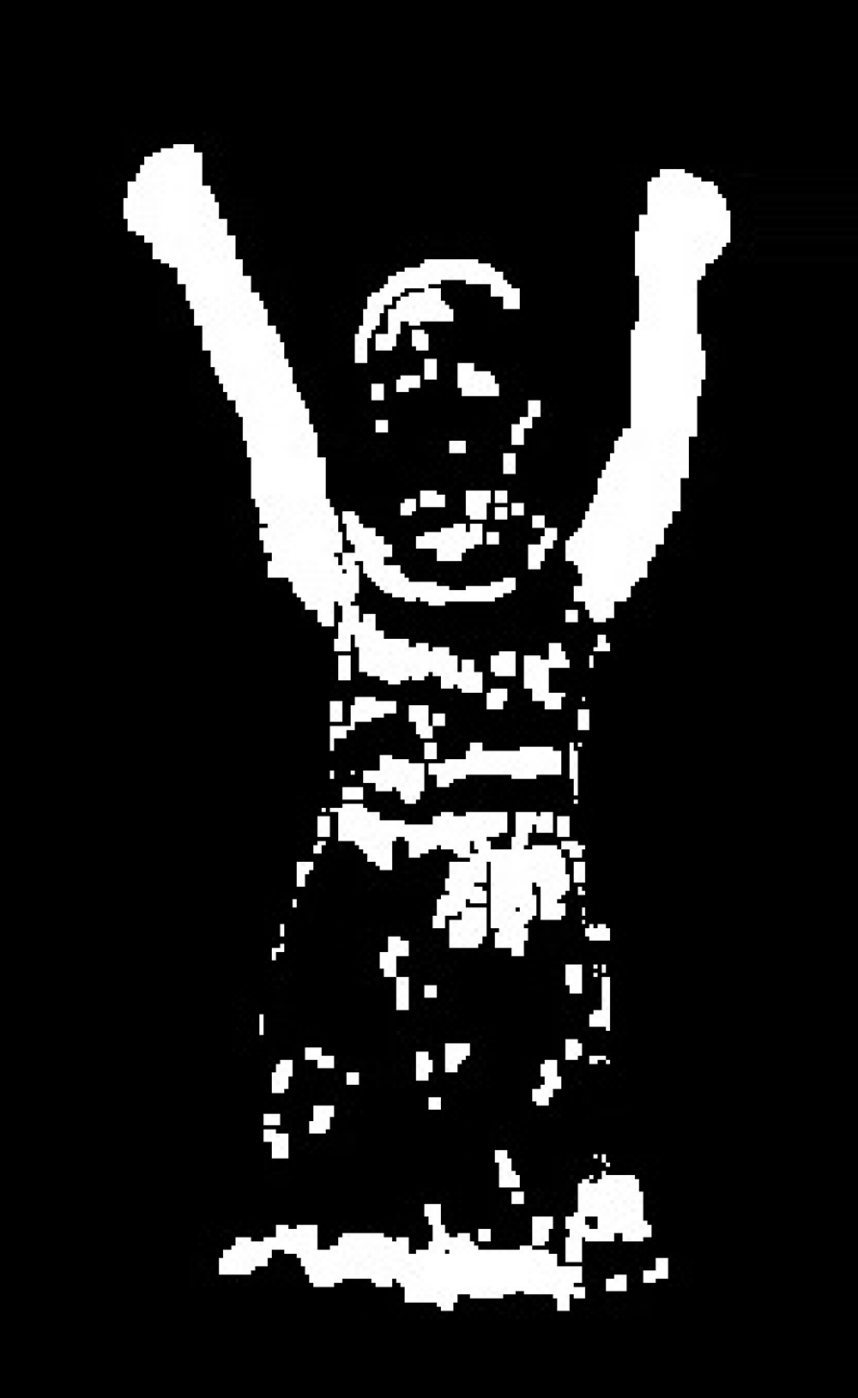
The Photograph of RGB Behavior Video Background Subtraction.

We adopt the ViBe algorithm improved with the above parameters. The frame of video unrelated background subtraction can be seen in Fig 2. Compared with other background subtraction methods, ViBe possesses many advantages as low memory usage, pixel processing and high anti-noise performances.

**Fig 3:**
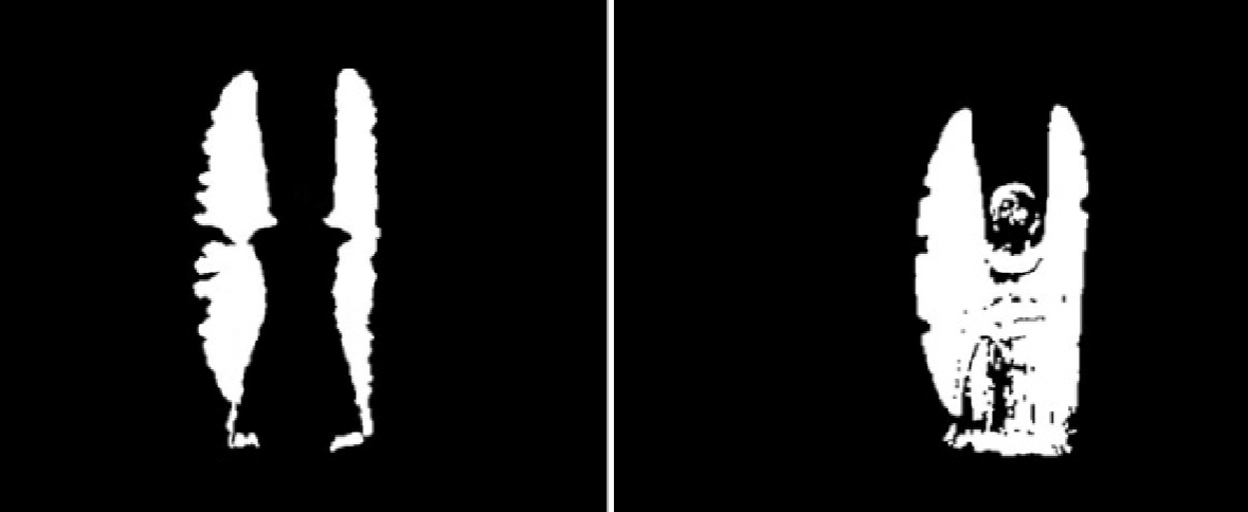
Behavior Motion Outline Model History Images of Depth Image (left) and RGB Image (right).

After the irrelevant background has been removed, human activity video is now becoming a binary video sequence without color component. Then, we compute the maximum pixel value for each pixel in each frame for description of Max Pixel Outline of the History Behavior Binary Image (MPOHBB). However, the MPOHHH of RGB video sequences obtained from method above still have residual noise pixels which is inevitable. But depth video itself has low sensitivity to space distance. We apply the same method to the depth video and obtain MPOHBB of depth video, which can ignore the inference of shake movement of people’s clothing. As for activity video of frames, the definition of MPOHBB is as Eq (2),

$$\begin{array}{c} I_{\max}=Max\{P(x,y,n),n=1,2,...,N\} \end{array}$$ (2)

where *P*(*x*, *y*, *n*) refers to the pixel value of the *n* frame video image in the (*x*, *y*) coordinate position, and *I*_*max*_ stands for the maximum binary pixel behavior outline history image. The behavior motion outline model history image on local behavior video database is shown in Fig 3.

### Marginalized processing of behavior model image

**Fig 4:**
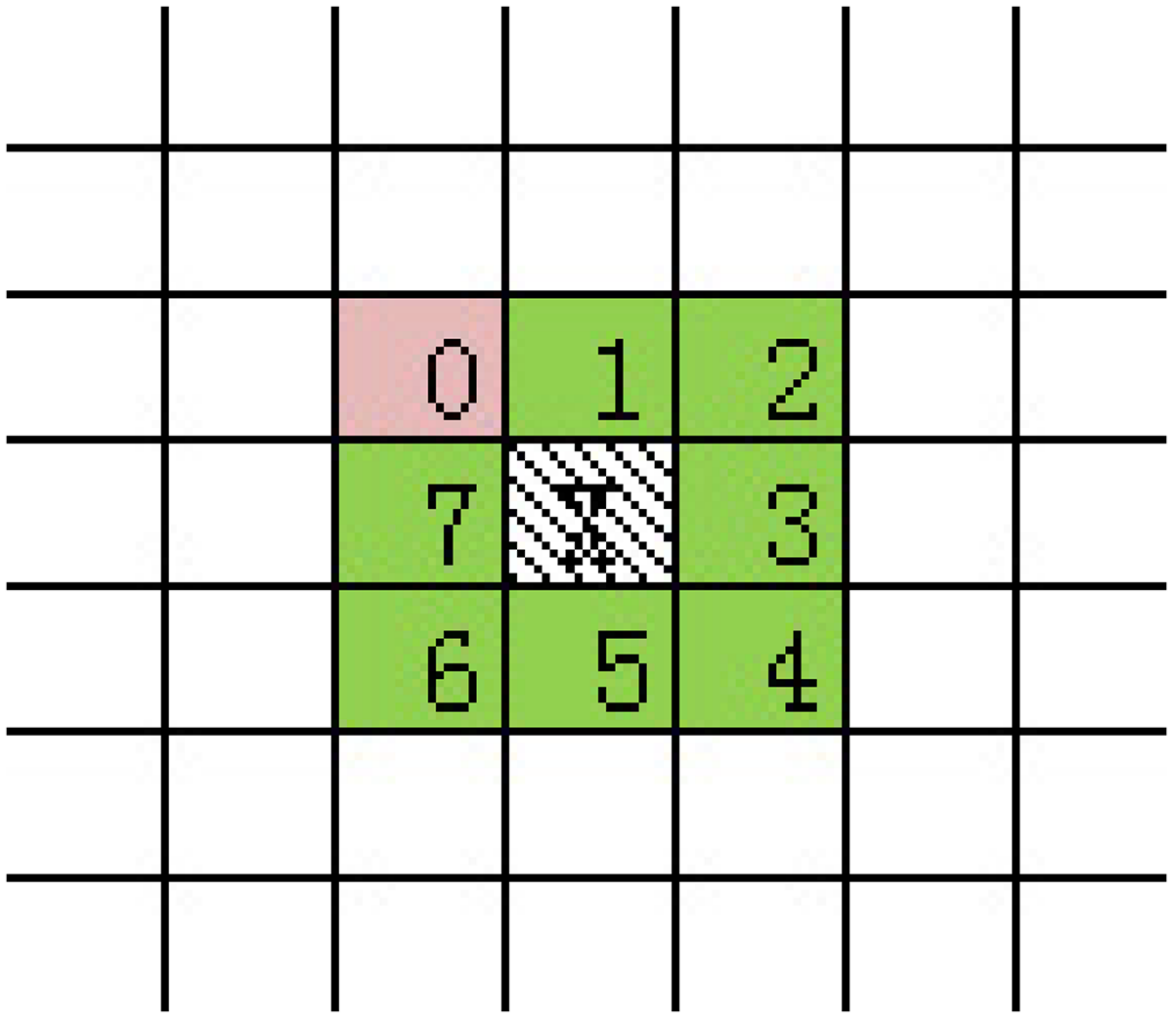
Behavior Motion Outline Model History Images of Depth Image (left) and RGB Image (right).

Based on the MHI of human activity in last subsection, we can see that Bump pixels exist due to lack of smoothing the edge pixels. So the step of smoothing the edge pixels is needed. First, we need to classify the edge pixels and enhance some pixels for better description. We define a special edge pixel, which surrounded 8 pixels *w*_0_ *w*_1_ *w*_2_…*w*_7_ are listed as follow clockwise in Fig 4.

**Fig 5:**
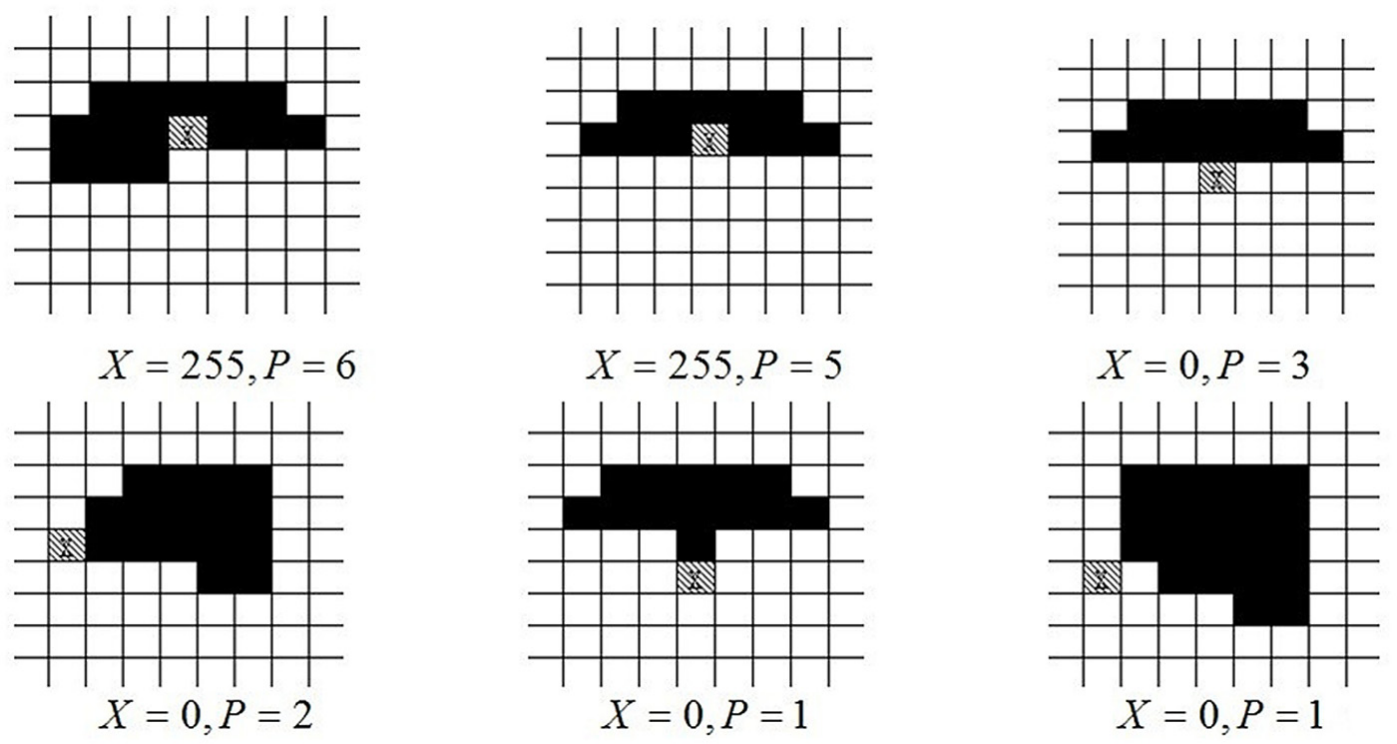
Category of Special Edge Pixel Circumstances.

Rotation-Invariant Local Binary Pattern (*B* = 8) is introduced to the binary sequences of *w* = (*w*_0_ *w*_1_ *w*_2_…*w*_7_)_2_, where *P* refers to the number of pixels which are continually unequal to *X* (0 ≤ *P* ≤ 8). Pixel value of 255 is defined as white, value of 0 is defined as black and other special edge pixel points circumstances refer to Fig 5.

According to the above special edge pixel circumstances, marginalized smooth processing is conducted and the processing method is as follow:

$$\begin{array}{c} V_{P}\left(X\right)=\{\begin{array}{c} 255-X,P=1,2,6\;(0,7,8\;non-edge\;pixel)\\ X,\;\;\;\;\;P=\;4\;\;(\;3,5\;only\;above) \end{array} \end{array}$$ (3)

**Fig 6:**
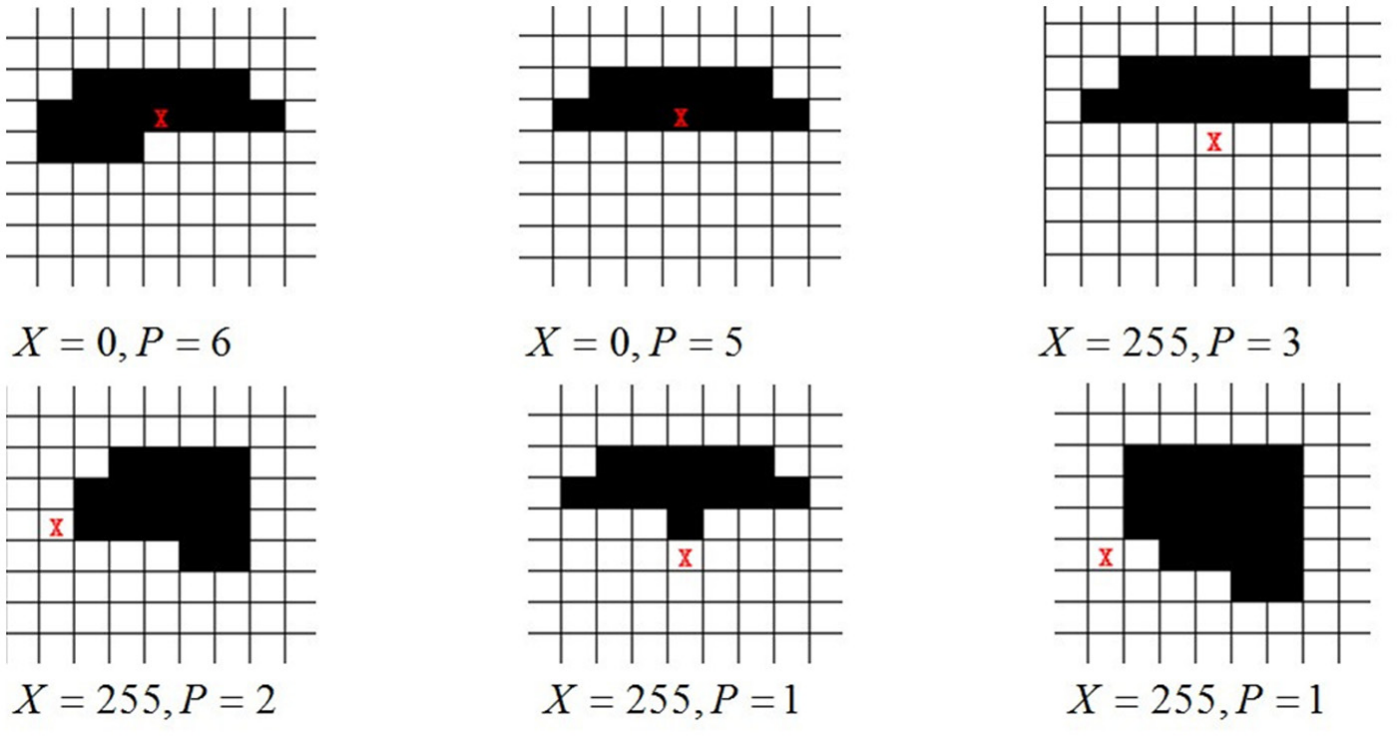
Result of Marginalized Processed Special Edge Pixels.

**Fig 7:**
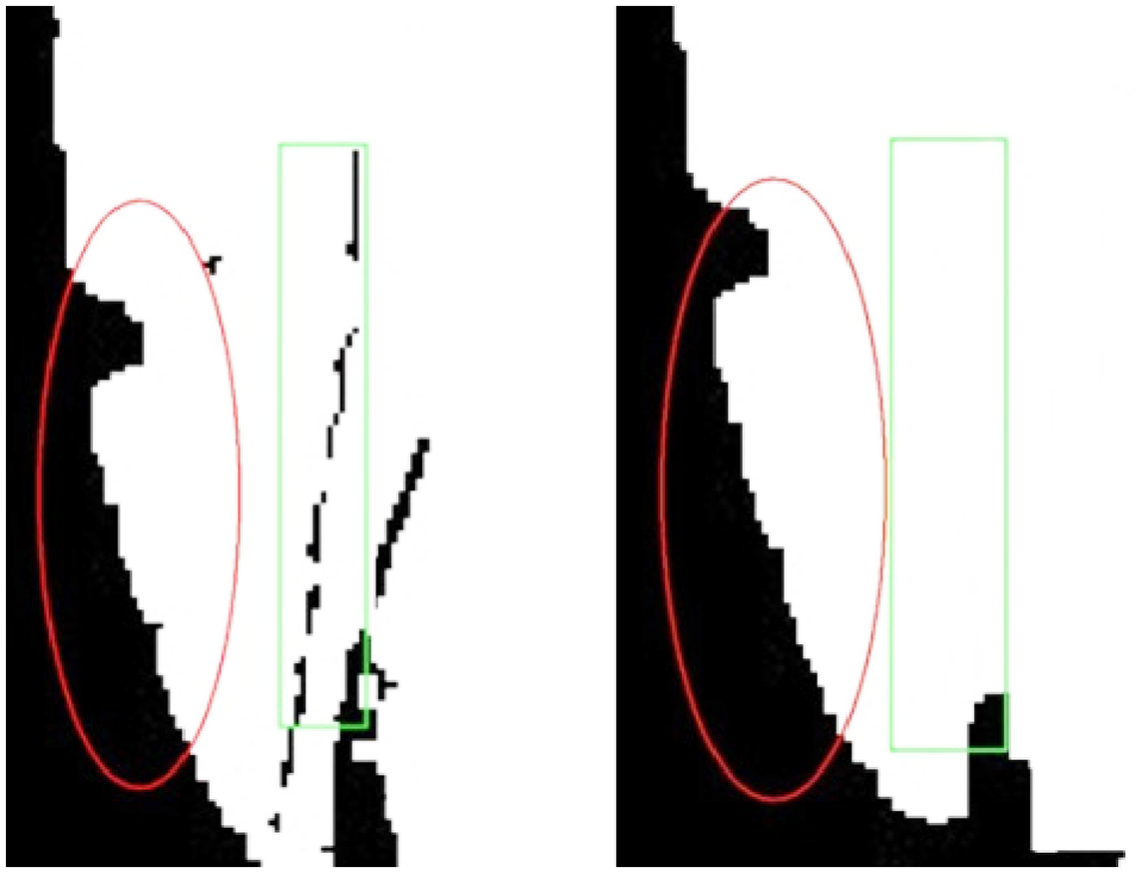
Contrast Photographs of Some images before (left) and after (right) Marginalization.

We smooth the edge in MHI of one single person by consecutive iteration. With more degree of iterations, the heavier the computation it takes, which is disadvantage to keeping the original characteristics of edges. We set *Iteration* = 3. The result of marginalized smoothing process is shown in Fig 6. The results of before-and-after marginalized processing are presented in Fig 7.

### RGB & Depth model image blending and blocking

Since the depth video only contains the depth information without RGB information, the fusion of depth information can well to remove residue and noise pixels. We propose to obtain the extraction of MHI from RGB and depth videos respectively and fuse the image in pixel level. The fusion follows two principles, which are to remove irrelevant edge pixels and interferential activity pixels as much as possible and keeping uttermost edge of MHI uttermost. *P*_*rgb*_(*x*, *y*) represents the pixel value of RGB videos generated history outline model image at the pixel (*x*, *y*). *P*_*depth*_(*x*, *y*) stands for the pixel value of depth video generated history outline model image at the pixel (*x*, *y*). *P*_*mix*_(*x*, *y*) refers to the pixel value of blended history outline model image at the pixel (*x*, *y*). To satisfy the above two principles, this paper proposed a simple blending methods as follow:

$$\begin{array}{c} P_{mix}\left(x,y\right)=\min\{P_{rgb}\left(x,y\right),P_{depth}\left(x,y\right)\} \end{array}$$ (4)

**Fig 8:**
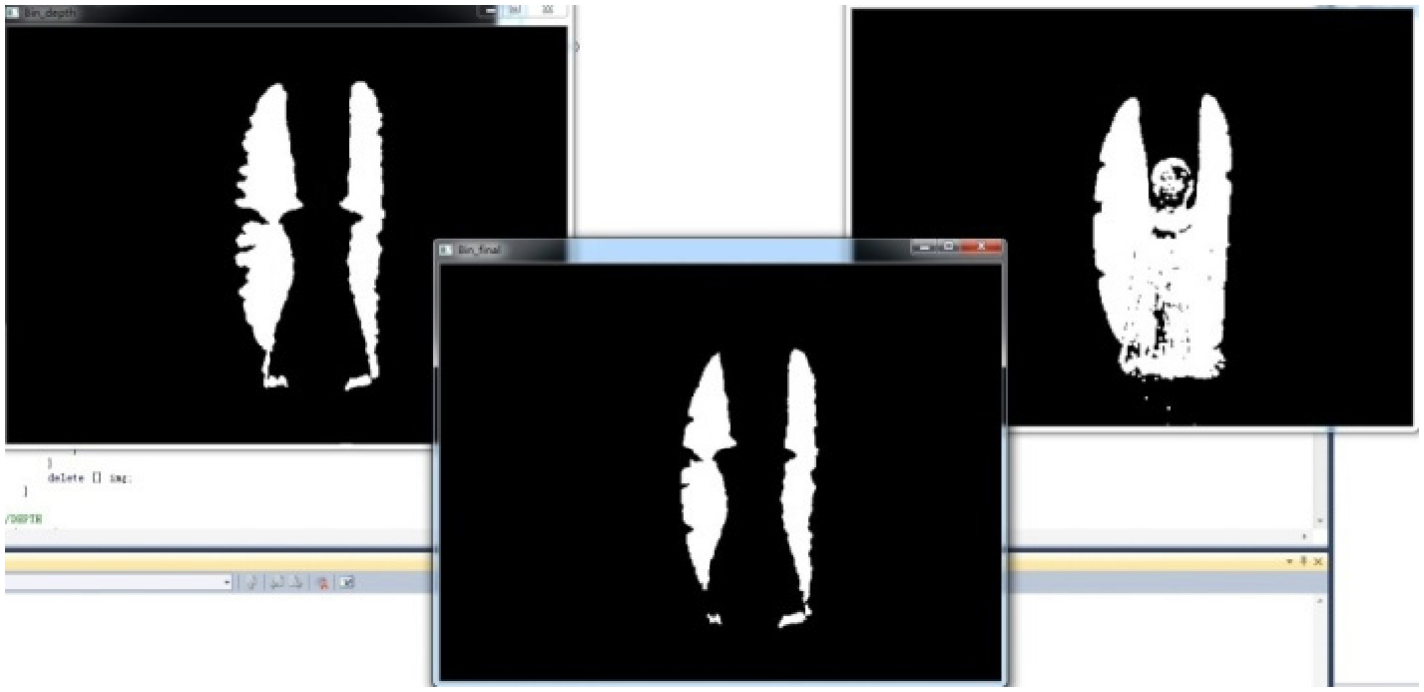
History Behavior Outline Image of Blended Depth and RGB Images.

Here, 0 ≤ *x* < *width*,0 ≤ *y* < *heigh*. Compared with the outline edge before blending, the blended history behavior outline model images are further strengthened and can better describe the outline features of the behaviors and reduced the interfering pixels of non-behavior outlines, which benefit the feature extraction backwards, as shown in Fig 8.

To further minimize the impact of background and noise on the image edge, the adjacent pixels in the outline images of motion behavior are divided into segments. The formula is as following:

$$\begin{array}{c} \bar{P_{\left(m,n\right)}}=\frac{1}{G^{2}}\sum_{i=0}^{G-1}\sum_{j=0}^{G-1}img((nG+i)width+(mG+j)\;)\\ \;\;Img\_block\left(m,n\right)=\{\begin{array}{c} 255\;,\;\;\bar{P_{\left(m,n\right)}}\geq T\\ \;\;0\;\;\;,\;\;\bar{P_{\left(m,n\right)}}<T \end{array} \end{array}$$ (5)

In this formula, *img*(*z*), *z* = *y* × *width*+*x* is the pixel value of historical behavior outline image on point (*x*, *y*). *G* × *G* is the size of segment. $\bar{P_{\left(m,n\right)}}$ is the average pixel value of each *G* × *G* segment. *Img*_*block*(*m*, *n*) is the pixel value of the pixel (*m*, *n*) generated after the division. *T* is the united discrimination threshold of *G* × *G*’s average pixel. The global threshold division approach is used in this paper, which means *T* = 128.

**Fig 9:**
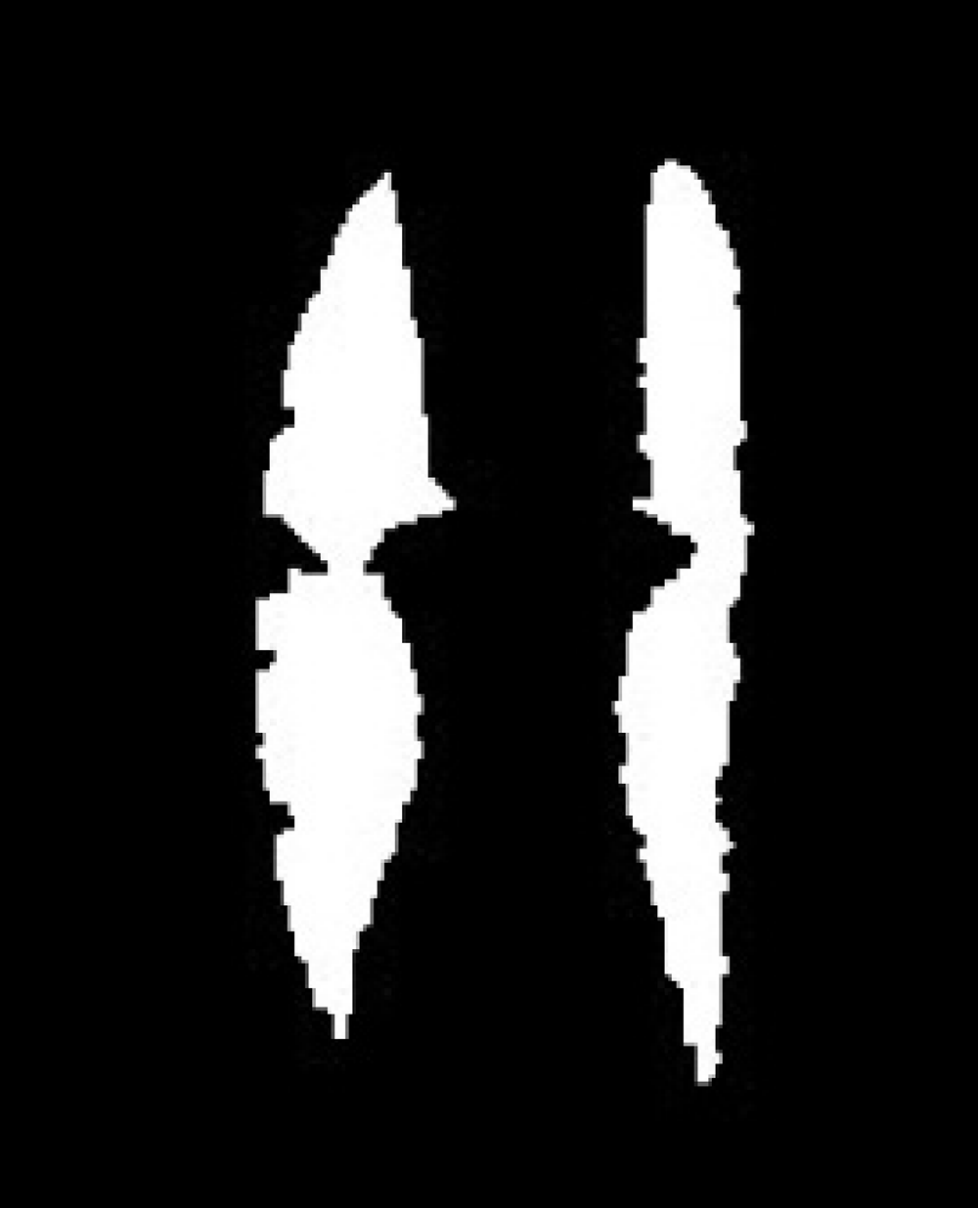
History behavior outline image integrated with depth and RGB images after 4 × 4 division.

In this paper, features extraction and classification are carried out on the divided and united images, and experiments show that its recognition rate is higher than the feature extraction of original images. This new algorithm reduces the influence of background pixels and cuts down the calculation amount of feature data by dividing the image into segments of same sizes (e.g. 1 × 1, 2 × 2, 4 × 4, 8 × 8, 10 × 10, 20 × 20). Moreover, it has been verified by the experiments that the segmentation effects 4 × 4 is better than other sizes. Thus, 4 × 4 segmentation pattern is adopted. The historical behavior outline image integrated with depth and RGB images after the division process is shown in Fig 9.

## Feature extraction and classification

In feature extraction aspect, this paper has robust distinguish ability to MHI that fuse depth and color images, which is achieved by the fusion of edge-texture feature that combines the LBP texture feature and edge feature. However, conventional feature extraction, which contains complicated and lower-speed procedure large amount of information, mostly employ SURF, SIFT or MoSIFT feature [27], to the disadvantage of upper classification and recognition. Local Binary Pattern (LBP) [35] is designed to describe the local texture feature of images, and it has been successfully applied in many technological areas. But traditional local binary patterns features target at texture description, it is hard to describe the change of the edge of the single texture. So we connect the characteristics of textures and edges, bringing the description method based on edge-texture features. In this paper, LBP is employed in the recognition of 3D human behaviors and acquires excellent recognition effects.

### RILBP, LBP^u2^, LBP^riu2^ Patterns feature

In most practical cases, researchers prefer to use Rotation-Invariant Local Binary Pattern (RILBP)[36] in accordance with the LBP to enhance the robustness of the rotation and shift of images. However, the former lacks of strong classification capacity. Among all the 2^*B*^ patterns of LBP, some patterns present quite low occurrence frequency in the images, while the proportions of some texture patterns are really high. Therefore, it can be regarded as the essential attribute of texture and all these patterns are called as Uniform Local Binary Pattern, noted as LBP^u2^ [37]. In the binary encoding of LBP, there are at most two shifts from 0 to 1. In this way, the histogram data of LBP can be degraded from 2^*B*^ to *B*(*B*−1)+2. LBP^u2^ has excellent classification capacity, and it can describe the vast majority of texture features.

Uniform Local Binary Pattern can also be created on the basis of RILBP, namely Rotation-Invariant Uniform Local Binary Pattern (noted as LBP^riu2^) [36]. Thus, all the LBP^u2^ values can be obtained by counting the number of “1” in binary encoding. Furthermore, its histogram data can be degraded to *B*+1, which reduces the feature data immensely.

### TE-LBP Pattern feature

**Fig 10:**
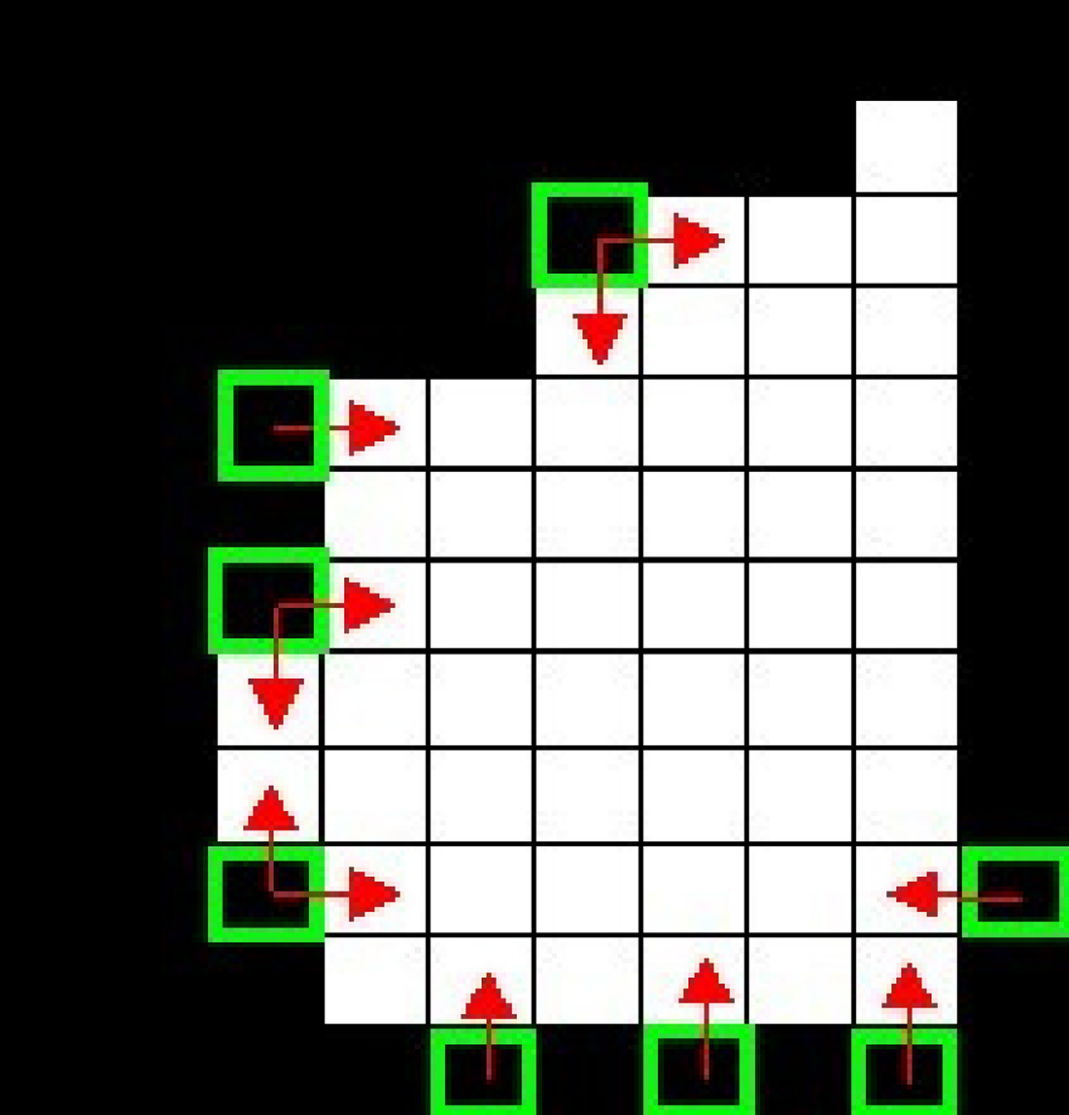
Gradient Vectors of Adjacent Edge Pixels.

In order to better characterize the texture and edge features of historical behavior outline image, we introduce a new algorithm integrated with texture and edge features in line with LBP, and it is named as Texture-Edge Local Binary Pattern (TE-LBP). The first thing is to define the gradient vector of adjacent edge pixels. Check the four adjacent directions of the central pixel (*x*, *y*). If the pixel value is larger than this central pixel exists, it is considered that the central pixel (*x*, *y*) has adjacent edge gradient vector $\vec{I_{x,y}}$, and four directions (up, down, left and right) are noted as $\vec{I_{u}},\vec{I_{d}},\vec{I_{l}},\vec{I_{r}}$. As shown in Fig 10, the module is the unit 1 and $\vec{I_{x,y}}=\vec{I_{u}}+\vec{I_{d}}+\vec{I_{l}}+\vec{I_{r}}$. When the edge pixel is processed with specific edge smoothing technology in previous Section, the edge pixel has at most two adjacent edge gradient vectors of two adjacent directions. As for the edge gradient vector of non-edge pixels, their modules are 0. Therefore, the features of non-edge pixels should be removed, while the features of edge pixels reserved. The definition of TE-LBP is as following:

$$\begin{array}{c} \mathrm{TE}-\mathrm{LBP}\left(i\right)=\sum_{x=0}^{W}\sum_{y=0}^{H}\;∥\vec{I_{x,y}}∥•\delta \left(\mathrm{LBP}\left(x,y\right),i\right)\;\\ \;\;\;\delta \left(m,n\right)=\{\begin{array}{c} \;1\;,\;m=n\\ \;0\;,\;m\neq n \end{array} \end{array}$$ (6)

where the data dimensions are 2^*B*^ and 0 ≤ *i* ≤ 2^*B*^.

### TE—LBP^u2^ Pattern feature

Since LBP^u2^ has a good classification capacity, we propose an integrated algorithm by combining texture with edge features on the basis of LBP^u2^. This algorithm is named as Texture-Edge Uniform Local Binary Pattern (TE—LBP^u2^). It can minimize the dimensions of feature data while taking the texture information of images into consideration. The definition of TE—LBP^u2^ is as following:

$$\begin{array}{c} \mathrm{TE}-\mathrm{LB}P^{u2}\left(i\right)=\sum_{x=0}^{W}\sum_{y=0}^{H}\;∥\vec{I_{x,y}}∥•\delta \left(\mathrm{LB}P^{u2}\left(x,y\right),i\right)\;\\ \;\;\;\delta \left(m,n\right)=\{\begin{array}{c} \;1\;,\;m=n\\ \;0\;,\;m\neq n \end{array} \end{array}$$ (7)

where the feature data directions are *B*(*B*−1)+2 and 0 ≤ *i* ≤ *B*(*B*−1)+2, and the definition of $\vec{I_{x,y}}$ is the same as TE-LBP.

### Classification algorithms

The most commonly used algorithms for human behavior recognition and classification are K-means based on the nearest neighbor, K-Nearest Neighbor (KNN) [38], Hidden Markov Model (HMM) [39], Dynamic Bayesian Network (DBN) [40], Conditional Random Fields Model (CRF) [41], etc. Among them, K-means based on the nearest neighbor and KNN are widely applied for their concise and fast classification properties. Given the small amount of sample data in this study, K-means based on the nearest neighbor and KNN are chosen as the classification algorithms, and the comparative analysis of their performances is carried out.

## Experimental results and discussion

In order to verify the feasibility of the behavior recognition algorithm proposed in this paper, experiments will be carried out in several public and private RGB and depth behavior data sets such as Weizmann Human Behavior Datasets, DHA Data Set and Local Behavior Data Set.

### Weizmann human behavior datasets

Since this data set only has RGB video sequences, the feasibility test of this paper is about the recognition and verification of its RGB video behaviors. The tested RGB video behaviors consist of 10 different behaviors, and each behavior will be completed by 9 people, respectively. According to Leave-one-out method [11], all the samples will be divided into *M* parts on the assumption that there are *M* people in total. We take out one part as the test set of behavior sample and leave the remaining 8 parts as training sets. Then, we change the test set in turn and use the remaining samples as new training sets. Repeat this trail for 9 times. Finally, we calculate their average recognition rate and take the result as the performance evaluation of this algorithm. Feature extraction will be conducted with TE—LBP^u2^ pattern. The recognition results are shown in Table 1 and Table 2.

**Table 1:** Confusion matrix of human behavior recognition of K-means.

Accuracy(%)		Train dataset									
		bend	jack	jump	pjump	run	side	skip	walk	wave1	wave2
Test dataset	bend	100									
	jack		100								
	jump			66.7		22.2		11.1			
	pjump				100						
	run			10.0		80.0		10.0			
	side						88.9		11.1		
	skip					10.0		90.0			
	walk						10.0		90.0		
	wave1									100	
	wave2										100

**Table 2:** Human behavior recognition of different classification algorithms.

Classification algorithms	K-means	KNN
Overall(%)	91.56	86.45

The recognition results show that it is difficult to identify actions with high similarity, such as “side”, “walk”, “run”, etc. However, only the features of RGB videos are extracted in this experiment, and it lacks of depth video. With an average recognition rate of 91.5%, this experiment is able to verify the feasibility of this paper’s application for RGB video behavior recognition. Besides, it can achieve good partition degree and recognition results.

### DHA behavior datasets

**Fig 11:**
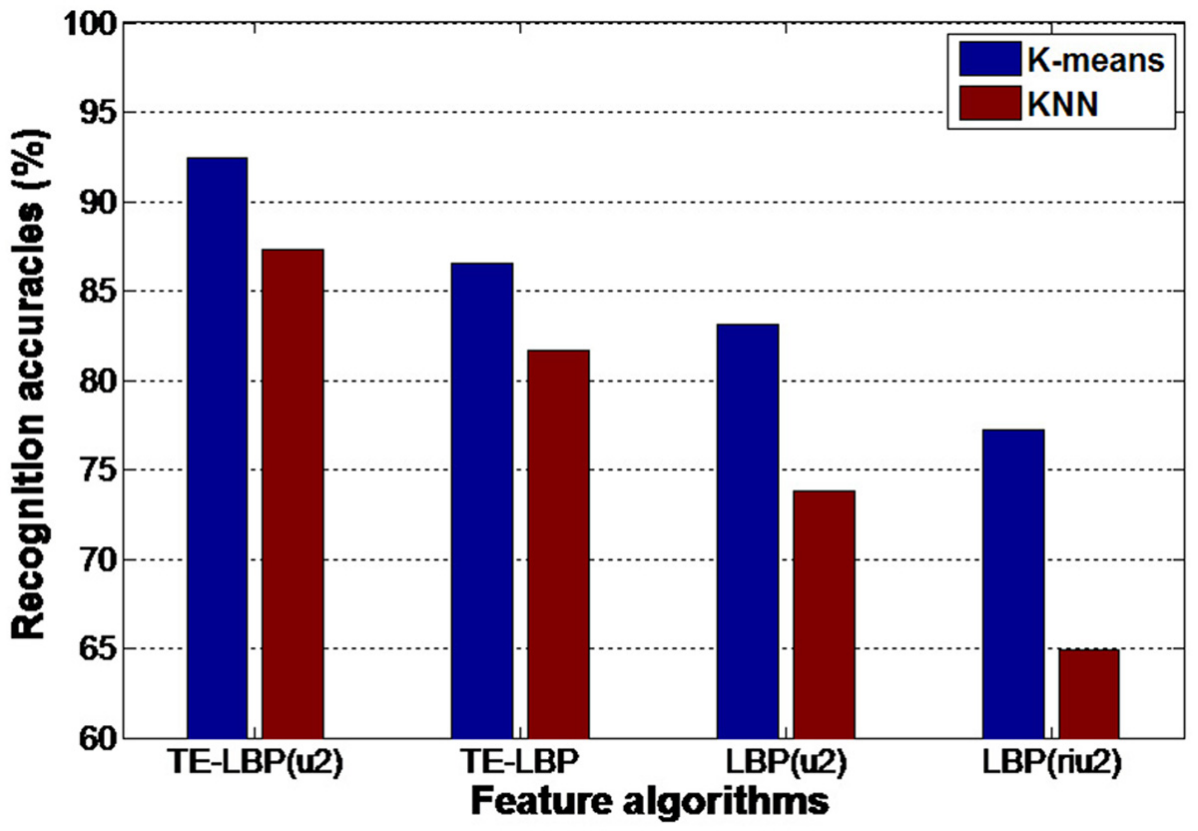
Recognition accuracies of four algorithms experiment on DHA behavior datasets.

This data set has 17 kinds of human behaviors and each behavior is accomplished by 21 different people. Obtained with Kinect-Like, this data set includes depth video sequences as well as RGB video sequences. Leave-one-out method is also applied in this experiment. All the samples are divided into 21 parts. We take out one part as the test set of behavior sample and leave the remaining 20 parts as training sets. Then, we change the test set in turn and use the remaining samples as new training sets. Finally, we calculate their average recognition rate and evaluate its performance. Feature extraction is conducted with TE—LBP^u2^, TE—LBP, LBP^u2^ and LBP^riu2^. Classification algorithms are K-means and KNN. The recognition results are shown in Fig 11.

It can be seen from the column diagram that the recognition rate is remarkably improved after the introduction of edge characteristic, and the effect of linear classification KNN is weaker than the effect of K-means cluster and classification algorithms. The results show that the recognition mechanism that integrates TE—LBP^u2^ with K-means algorithm has higher recognition rate than other features in this data set. Its recognition rate is above 92%, which shows certain improvements compared with previous researches. Consequently, the experimental results prove that this recognition algorithm has excellent effects on behavior recognition of 3D videos solidly.

### Local human behavior datasets

**Fig 12:**
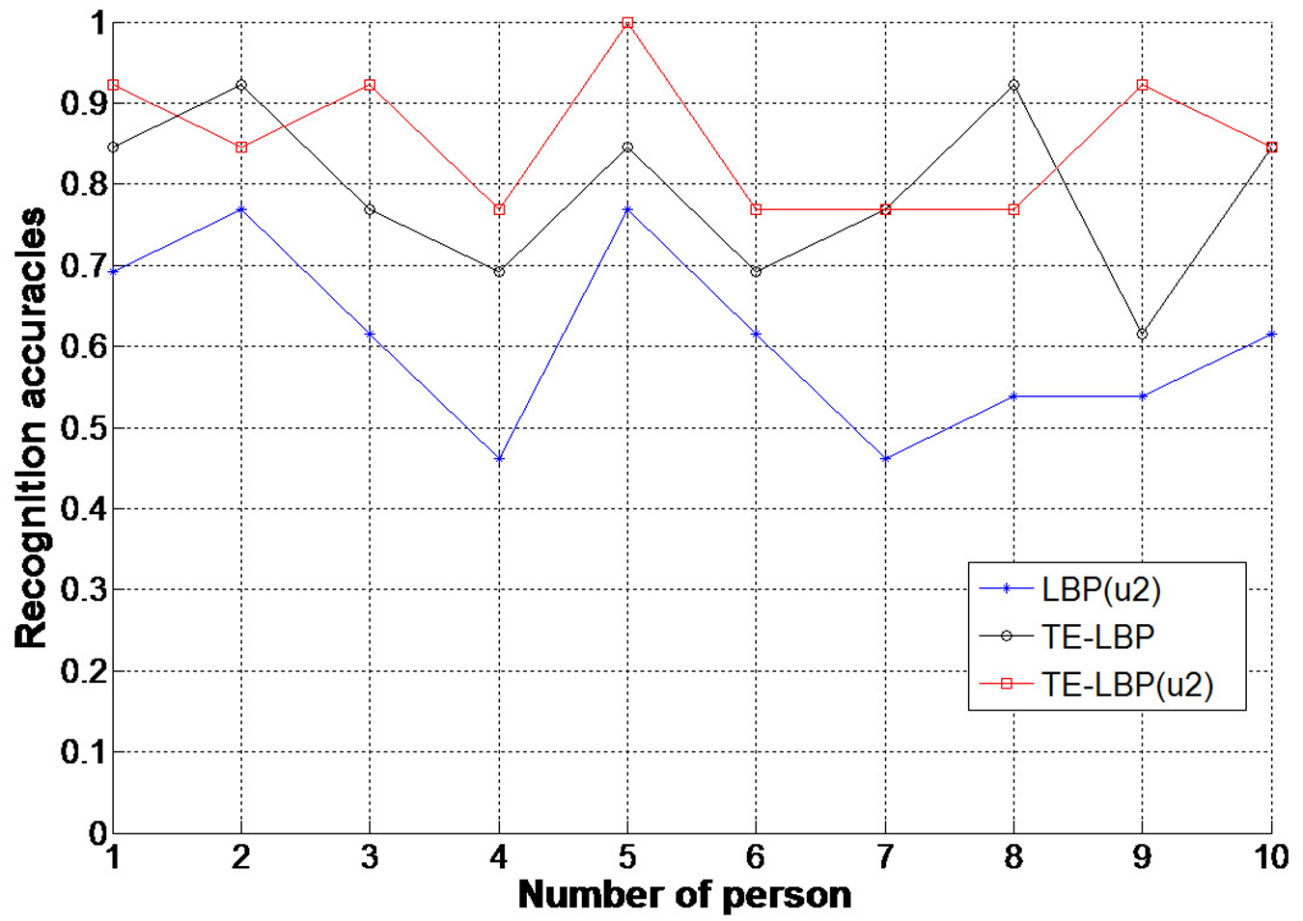
Recognition accuracies of three algorithms experiment based on K-means on Local behavior datasets.

This data set is collected by the local laboratory with Kinect somatosensory devices. It also has both RGB and depth video sequences. It is made up of 13 kinds of human behaviors and each of them will be accomplished by 10 people. All the samples will be separated into 10 parts in line with Leave-one-out method. We take out one part as the test set of behavior sample and leave the remaining 9 parts as training sets. Then, we change the test set in turn and use the remaining samples as new training sets. Finally, we calculate their average recognition rate and evaluate its performance. Feature extraction is conducted with TE—LBP^u2^, TE—LBP, LBP^u2^ and LBP^riu2^. Classification algorithms are K-means and KNN. The recognition results in local 3D human behavior data set are shown as Fig 12 and Table 3.

**Table 3:** Human behavior recognition of different classification algorithms.

Classification algorithms	KNN	K-means
LBP^u2^	54.61%	60.77%
*TE*−*LBP*	70.77%	79.23%
TE—LBP^u2^	81.53%	85.38%

**Fig 13:**
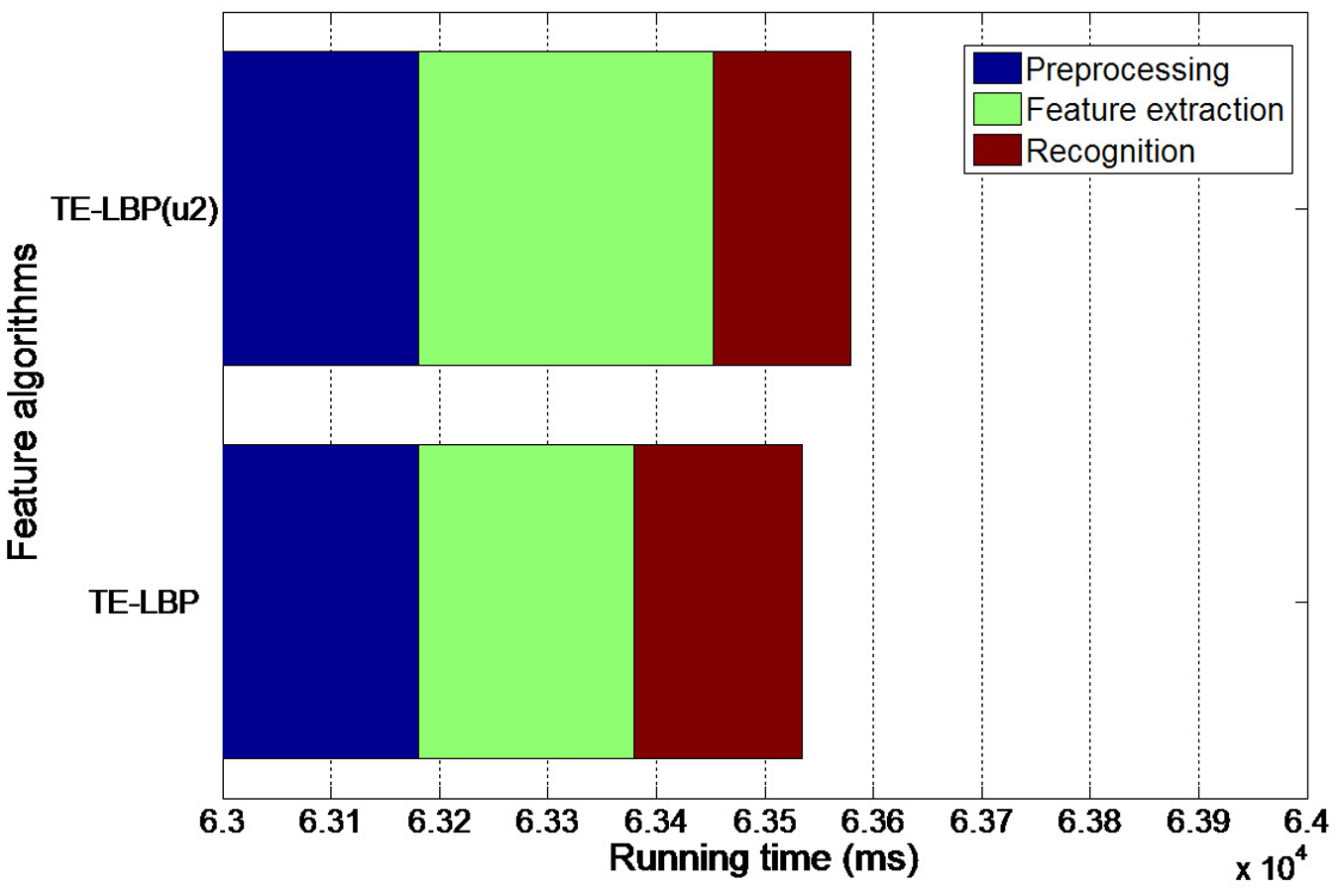
Running time of two feature algorithms.

The recognition algorithm of this paper obtains excellent results in the local behavior video data set. According to Fig 12, the recognition rate of TE—LBP^u2^ is much higher than TE-LBP and LBP^u2^, which indicates that texture feature merged with edge characteristic has stronger robustness for 3D human behavior videos. Although the recognition rate is lower than the public behavior video databases, which makes this algorithm become more challenging. Table 3 indicates that the results of K-means cluster and nearest neighbor classification are better than direct KNN classification. For this reason, K-means is applied in this paper. In the same pair of behavior videos, the pre-processing runtime, feature extraction time and classification recognition time of their TE-LBP and TE—LBP^u2^ features are tested, and detailed results are described in Fig 13. When compared with TE-LBP, TE—LBP^u2^ has longer extraction time and shorter classification recognition time with higher recognition rate. In general, the algorithm proposed in this paper has relatively smaller image feature data volume after pre-processing when compared with other behavior recognition algorithms. As a result, this algorithm reduces the runtime and improves the recognition speed. Experiments prove that this algorithm can be widely applied in human behavior recognition of 3D depth videos.

## Conclusions

In this paper, a human behavior recognition algorithm is introduced by integrating TE—LBP^u2^ feature with 3D depth videos. In comparison with the recognition algorithms of previous researches, this algorithm greatly reduces the data volume of features. It also enhances the recognition speed with modified local pattern extraction feature algorithm. Moreover, this algorithm achieves excellent recognition rate and has strong robustness against external interference factors. It simplifies the recognition process of video human behavior recognition to a certain extent and is easy to operate.

To sum up, this algorithm has certain feasibility and reasonability if the identified human behaviors have large differences. The following research will be focused on the optimization of video pre-processing and improvements of feature extraction algorithm; the loss of motion features data should be minimized and recognition rate should be enhanced while taking recognition speed into account.
